# Interplay between systemic inflammation, anemia, and mycobacterial dissemination and its impact on mortality in TB-associated HIV: a prospective cohort study

**DOI:** 10.3389/fimmu.2023.1177432

**Published:** 2023-04-18

**Authors:** Mariana Araújo-Pereira, Charlotte Schutz, Beatriz Barreto-Duarte, David Barr, Klauss Villalva-Serra, Caian L. Vinhaes, Amy Ward, Graeme Meintjes, Bruno B. Andrade

**Affiliations:** ^1^ Instituto Gonçalo Moniz, Fundação Oswaldo Cruz, Salvador, Brazil; ^2^ Multinational Organization Network Sponsoring Translational and Epidemiological Research (MONSTER) Initiative, Salvador, Brazil; ^3^ Programa de Pós-Graduação em Patologia Humana e Experimental, Universidade Federal da Bahia, Salvador, Bahia, Brazil; ^4^ Curso de Medicina, UNIFTC, Salvador, Bahia, Brazil; ^5^ Department of Medicine, University of Cape Town, Cape Town, South Africa; ^6^ Wellcome Centre for Infectious Diseases Research in Africa (CIDRI-Africa), Institute of Infectious Disease and Molecular Medicine, Faculty of Health Sciences, University of Cape Town, Cape Town, South Africa; ^7^ Programa de Pós-Graduação em Clínica Médica, Universidade Federal do Rio de Janeiro, Rio de Janeiro, Brazil; ^8^ Curso de Medicina, Universidade Salvador (UNIFACS), Salvador, Bahia, Brazil; ^9^ Department of Infectious Diseases, NHS Greater Glasgow & Clyde, Glasgow, United Kingdom; ^10^ Bahiana School of Medicine and Public Health, Bahia Foundation for the Development of Sciences, Salvador, Brazil

**Keywords:** anemia, HIV, tuberculosis, mortality, systemic inflammation

## Abstract

**Introduction:**

Anemia frequently affects people living with HIV (PLHIV). Nevertheless, the impact of anemia on treatment outcomes of patients with HIV-associated tuberculosis (TB) and the underlying molecular profiles are not fully characterized. The aim of this study was to investigate the interplay between anemia, the systemic inflammatory profile, dissemination of TB and death in HIV-TB patients in an ad hoc analysis of results from a prospective cohort study.

**Methods:**

496 hospitalized PLHIV ≥18 years old, with CD4 count <350 cells/μL and high clinical suspicion of new TB infection were enrolled in Cape Town between 2014-2016. Patients were classified according to anemia severity in non-anemic, mild, moderate, or severe anemia. Clinical, microbiologic, and immunologic data were collected at baseline. Hierarchical cluster analysis, degree of inflammatory perturbation, survival curves and C-statistics analyses were performed.

**Results:**

Through the analysis of several clinical and laboratory parameters, we observed that those with severe anemia exhibited greater systemic inflammation, characterized by high concentrations of IL-8, IL-1RA and IL-6. Furthermore, severe anemia was associated with a higher Mtb dissemination score and a higher risk of death, particularly within 7 days of admission. Most of the patients who died had severe anemia and had a more pronounced systemic inflammatory profile.

**Discussion:**

Therefore, the results presented here reveal that severe anemia is associated with greater TB dissemination and increased risk of death in PLHIV. Early identification of such patients through measurement of Hb levels may drive closer monitoring to reduce mortality. Future investigations are warranted to test whether early interventions impact survival of this vulnerable population.

## Introduction

It is estimated that about 24.8% of the world population is affected by some type of anemia (1), being especially prevalent in immunocompromised patients (2). Anemia is defined by a decrease in hemoglobin (Hb) values below well-established cut-offs (<13 g/dL for men; and <12 g/dL for women) (3). The presence of anemia is closely associated with other pathologies and immuno-inflammatory statuses, such as malnutrition, micronutrient deficiencies, inflammation, and infectious diseases (3, 4).

Importantly, anemia is a very frequent comorbidity in people living with HIV (PLHIV), with prevalence ranging from 21 to 71% (5), and is associated with greater all-cause mortality (6), higher HIV viral load, lower CD4 count, and HIV disease progression (7, 8). The etiology of anemia in PLHIV is commonly attributed to chronic inflammation/disease (9), lower iron intake leading to iron deficiency, presence of co-infections, or could also be caused by certain antiretroviral drugs as a side effect (10, 11).

Anemia is also a prevalent finding in tuberculosis (TB) patients, ranging from 44 to 89% (12), and is related to higher rates of treatment failure. Of note, HIV is well described as a risk factor for the development of active TB. Considering that anemia is more frequently observed in PLHIV with advanced disease, it is possible that it may also be an underlying factor contributing to increased odds of progression from latent to active TB (13). HIV-associated TB (hereafter mentioned as HIV-TB) was responsible for more than 200,000 deaths in 2020 (13). Therefore, deciphering whether anemia in HIV-TB patients undermines prognosis during hospitalization through association with unfettered systemic inflammation and TB dissemination is critical to improve at least two aspects of clinical management. First, early identification of anemia could help identifying patients at higher risk of death. Lastly, the identification of the relationships between mycobacterial dissemination, a unique profile of systemic inflammation and anemia severity could uncover immune mechanisms underlying increased mortality.

The present study investigated the impact of anemia on the inflammatory profile in a cohort of hospitalized persons with HIV-TB from South Africa. We also linked the severity of the anemia to important clinical features on presentation and outcomes, such as dissemination of *Mycobacterium tuberculosis* (*Mtb*) and death, respectively, in context of HIV-TB infection.

## Methods

### Ethical review of the study and informed consent of study participants

The study was approved by the University of Cape Town Human Research Ethics Committee (UCT HREC), reference number 057/2013. Participants provided written informed consent when possible. Eligible patients with a decreased level of consciousness were enrolled and followed up daily until they regained capacity to participate in the informed consent process, and if not agreeable to participate, were withdrawn from the study. The UCT HREC approved the use of information from participants who died prior to providing informed consent by the end of study follow-up.

### Study population and procedures

This study is a sub-analysis of a prospective observational cohort which recruited participants in Khayelitsha Hospital, Cape Town. Thus, this is a convenience sample. The study was conducted from January 2013 to October 2016 as previously described (14). PLHIV ≥18 years old, with CD4 count <350 cells/μL and a high clinical suspicion of new TB were eligible for enrolment. Pregnant women, history of anti-TB therapy within the last month, or those who were recently initiated and received three or more doses of anti-TB therapy were not eligible for enrolment (14). Detailed description of the cohort is found in a previous publication (14).

### Laboratory assays

Sputum TB cultures, sputum Xpert *Mtb*/RIF assay (Cepheid), urine Xpert *Mtb*/RIF assay, *Mycobacterial* blood culture and the GenoType *Mtb*DRplus assay (Hain Lifesciences) were performed at the National Health Laboratory Services (NHLS) and used to provide TB diagnosis. CD4 count, HIV viral load, full blood count, differential count, renal function, liver function, C-reactive protein (CRP), procalcitonin, venous lactate, and cytomegalovirus (CMV) viral load tests were performed on all participants by the NHLS, as previously reported. CMV was measured using the Argene CMV R-gene platform and a viral load >49 IU/mL was regarded as detectable (14).

Plasma was stored at −80°C for immunology assays. Soluble inflammatory mediators were tested on stored plasma (1:2 dilution) using Luminex technology (Bio-Plex Pro Human Cytokine Standard 27-Plex kit). The following analytes were measured: interleukin (IL)-1β, IL-1 receptor antagonist (IL-1Ra), IL-2, IL-4, IL-5, IL-6, IL-7, IL-8, IL-9, IL-10, IL-12p70, IL-13, IL-15, IL-17A, eotaxin, basic fibroblast growth factor (FGF), granulocyte colony stimulating factor (G-CSF)/colony stimulating factor 3 (CSF3), granulocyte-macrophage colony stimulating factor (GM-CSF/CSF2), interferon gamma (IFN-γ), interferon gamma-induced protein (IP-10)/C-X-C motif chemokine ligand 10 (CXCL10), monocyte chemoattractant protein-1 (MCP-1)/C-C motif chemokine ligand 2 (CCL2), macrophage inflammatory protein-1 alpha (MIP-1α/CCL3), MIP-1 beta (MIP-1β/CCL4), platelet-derived growth factor-BB (PDGF), regulated on activation, normal T cell expressed and secreted (RANTES/CCL5), tumor necrosis factor-alpha (TNF), and vascular endothelial growth factor (VEGF). For statistical analyses, mean fluorescence intensity (MFI) values of the plasma markers were used. Such approach allows for analysis of analytes of low abundance and does not require censoring or correction for background (14–16).

### Data collection and definitions

Clinical data were obtained from the patient’s hospital folder, and clinical review at enrolment and captured on standard case record forms. The primary outcome was vital status at week 12. Participants with a health system record entry indicating a clinic visit, collection of medication, or a laboratory test performed beyond week 12 were assumed to be alive at week 12.

Urine lipoarabinomannan (LAM) more than or equal to grade 1 by two independent readers was regarded as positive. “Microbiologically confirmed TB” was defined as participants with *Mtb* on at least 1 culture or Xpert *Mtb*/RIF test from any clinical sample. “Probable tuberculosis” was defined as participants without microbiologically confirmed TB who had positive urine LAM or had a compatible clinical and radiological picture and were treated for TB and without alternative primary diagnosis made during enrolment admission. Early deaths were deaths that occurred within 7 days of enrolment, and late deaths are all deaths that occurred after 7 days and within 12 weeks of enrolment.

### Anemia definitions

According to the World Health Organization (WHO) guideline criteria, anemia was defined as levels of Hb below 13 g/dL for men or <12 g/dL for women (3). Mild anemia was defined as Hb value >10 g/dL and <13 g/dL for men; and >10 and <12 g/dL for women, whereas moderate anemia was defined as Hb>8 g/dL and <=10 g/dL for both sexes. Severe anemia was defined as Hb<8g/dL for both sexes (**Supplementary Figure 5**) (3).

### Degree of mycobacterial dissemination

The degree of *Mtb* dissemination was defined with a three-point dissemination score, as previously described by our group (17). Participants were allocated 1 point for the following: urine LAM test positive, mycobacterial blood culture positive and identified as *Mtb* and urine Xpert *Mtb*/RIF assay positive for *Mtb*, yielding a score ranging from 0-3 (18).

### Inflammatory profile and degree of inflammatory perturbation

To evaluate the overall profile of systemic inflammation and how it related to degree of anemia, we log_10_ transformed the biomarker values and performed an unsupervised hierarchical cluster analysis (Ward’s method), with dendrograms representing the Euclidean distances. We calculated the Degree of Inflammatory Perturbation (DIP), which measures the overall level of inflammation in a patient by analyzing several biomarkers. DIP was calculated using data of all the available cytokines to identify the general inflammatory environment of the participants. DIP was adapted from the molecular degree of perturbation, which has been described previously (19). For this study, the DIP calculation included the concentrations of the plasma inflammatory markers instead of gene expression values in the original analysis model (19). Thus, herein, the average level and standard deviation of a baseline reference group (without anemia) were calculated for each biomarker. The DIP score of each biomarker was defined by z-score normalization, where the differences in concentration values from the average of the biomarker in reference group was divided by the reference standard deviation. Therefore, the DIP score represents the differences by number of standard deviations from the control group. Similar approaches resulting in DIP-like scores have been previously employed using biomarker measurements by our group (20, 21). We ranked the top 10 markers which contributed the most for the DIP score values (using the sum of DIP score for each variable in all test groups), to identify the most informative soluble mediators contributing to the overall inflammatory disturbance.

### Statistical analysis

Descriptive statistics were used to present data, and median values with interquartile ranges (IQR) were used as measures of central tendency and dispersion, for continuous variables. Only complete cases were evaluated. Categorical variables were described using frequency (no.) and proportions (%). The Pearson’s chi-square test was used to compare categorical variables between study groups. The Mann-Whitney *U* test (2 groups) or the Kruskal–Wallis test (>2 groups) were used to compare continuous variables. The Cochran–Armitage test for trend was used to assess for the presence of an association between the DIP levels and clinical characteristics with the severity of anemia.

Kaplan-Meier analysis was performed using the log-rank (Mantel-Cox) test and applied to estimate death probability of the participants stratified based on the hierarchical cluster. Differences with p-values below 0.05 after adjustment for multiple comparisons (Holm-Bonferroni) were considered statistically significant. The statistical analyses were performed using and R language (version 4.4.1).

## Results

### Characteristics of the cohort

This prospective cohort was composed of 659 hospitalized PLHIV and with suspected TB, enrolled a median of 2 days (IQR:1-3) after hospital admission. In our analysis, we included only confirmed or probable TB cases, excluding possible and not TB. We excluded patients without cytokines data and those lost to follow-up, resulting in 496 patients in the analysis (**Supplementary Figure 1A**).

The cohort was further stratified according to the occurrence and severity of anemia. We found that 7.3% (n=36) of the patients had normal Hb levels according to WHO definitions. The remaining 92.7% (n=460) had low Hb levels and were considered anemic. Comparing these two main groups, we found that anemic patients presented with lower weight (without anemia, median [IQR]: 57.0Kg [49.2-75.8]; with anemia: 54.0Kg [47.0-61.0]; p=0.025) and CD4 count (without anemia: 110 cells/µL [50-162]; with anemia: 55.0 cells/µL [20-111]; p=0.001) as well as higher frequency of CMV detectable in blood (without anemia: n=6, 16.7%; with anemia: n=186, 41%; p=0.007) and higher frequency with positive urine *Mtb* Xpert test (without anemia: n=7, 22.6%; with anemia: n=181, 40.6%; p=0.002) (**Supplementary Table 1**).

Next, we stratified the anemic patients according to the severity into mild (23.4%, n=116), moderate (31.2%, n=155), and severe (38.1%, n=189) (**Table 1**). We found a predominance of female sex among participants with severe anemia (without anemia: 41.7%; mild: 42.2%; moderate: 51.6%; severe: 62.4%; p=0.003). Importantly, a decrease of CD4 T-cell counts was detected according to anemia severity (without anemia: 110 cells/µL [50-162]; mild: 69 cells/µL [23.8-140]; moderate: 57 cells/µL [25.5-120]; severe: 42 cells/µL [17-101]; p<0.001) (**Supplementary Figure 1B**). Furthermore, a higher proportion of positive *Mtb* blood cultures was observed as anemia severity worsened (without anemia: 22.6%; mild: 33.3%; moderate: 36.4%; severe: 48.4%; p=0.014). The same phenomenon was observed for positive Urine *Mtb* Xpert tests (without anemia: 18.8%; mild: 32.0%; moderate: 48.1%; severe: 59.6%; p<0.001). That is, these parameters showed a tendency to increase (in the case of female frequency, CMV detection, *Mtb* blood culture and Urine *Mtb* Xpert positive result) or decrease (CD4 count) as anemia severity worsened (**Supplementary Figure 1B**, **Table 1**).

**Table 1:** Clinical characteristics of HIV-TB patients according anemia.

	Without anemia (n=36)	Mild anemia (n=116)	Moderate anemia (n=155)	Severe anemia (n=189)	p value
Sex (female), (%):	15 (41.7)	49 (42.2)	80 (51.6)	118 (62.4)	0.003*
Age (years), median (IQR):	37.6 (31.8-50.2)	37.8 (32.5-43.9)	35.8 (31.1-42.6)	35.1 (30.0-41.3)	0.046*
Weight (kg), median (IQR):	57.0 (49.2-75.8)	54.0 (49.0-60.8)	53.0 (46.4-61.0)	54.0 (47.0-62.0)	0.063
ART naive, n (%):	19 (52.8)	50 (43.5)	65 (41.9)	64 (33.9)	0.181
CD4 (count), median (IQR):	110 (50.0-162)	69.0 (23.8-140)	57.0 (23.5-120)	42.0 (17.0-101)	<0.001*
HIV (log_10_ copies/mL) VL, median (IQR)	5.25 (4.35-5.70)	5.28 (3.77-5.68)	5.18 (3.64-5.75)	5.15 (3.84-5.77)	0.953
Blood detected CMV, n (%):	6 (16.7)	44 (38.3)	61 (40.1)	81 (43.3)	0.028*
*Mtb* blood culture, n (%):	7 (22.6)	37 (33.3)	55 (36.4)	89 (48.4)	0.014*
Urine Xpert *Mtb*/RIF assay positive, n (%):	6 (18.8)	33 (32.0)	64 (48.1)	96 (59.6)	<0.001*

Bold font indicates statistical significance. Mild anemia was defined as Hb value >10 g/dL and <13 g/dL for men; and >10 and <12 g/dL for women, whereas moderate anemia was defined as Hb>8 g/dL and <=10 g/dL for both sexes. Severe anemia was defined as Hb<8g/dL for both sexes. The severity of anemia according to the Hb levels is defined in methods. Data are shown as median and interquartile (IQR) range or frequency (percentage). Categorical data were compared between the clinical groups using the Chi-squared tests. Continuous data were compared between the clinical groups using the Kruskall-Wallis (for more than two groups) or Mann-Whitney *U* test (for two unmatched groups). *Represent p <0.05 in Cochran–Armitage test for trend.

We further investigated in more details the relationships with Hb values and key clinical laboratory parameters. In this cohort, Hb values were weakly positively associated with CD4 counts (rho: 0.16; p<0.001) and not related with HIV viral loads (rho: -0.02; p=0.71) (**Supplementary Figures 2A, B**). As expected, CD4 counts were inversely correlated with HIV viral loads (**Supplementary Figure 2C**).

### Association between anemia severity, TB dissemination and death

**Figure 1:**
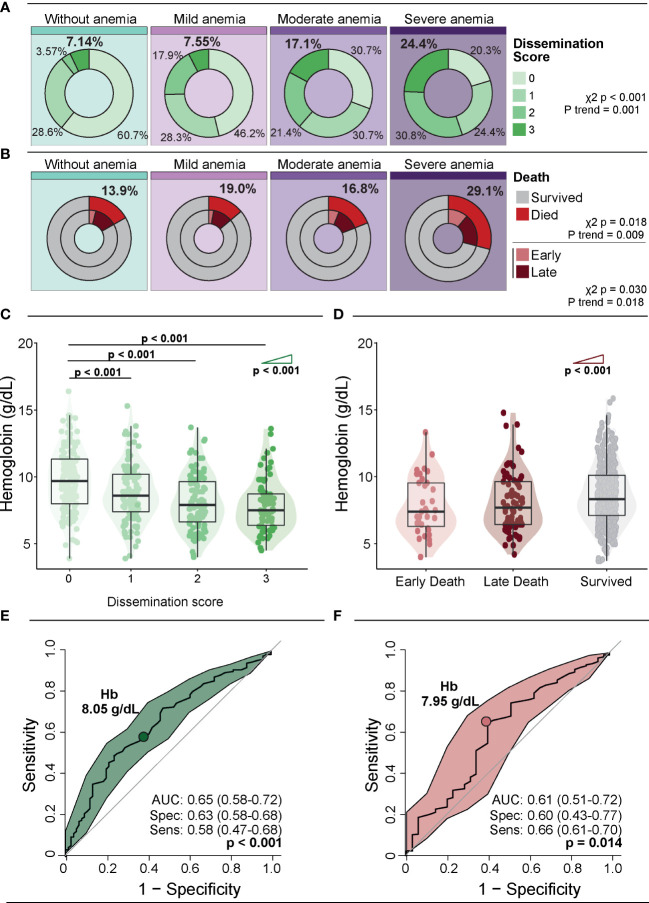
Association of anemia severity with Mtb dissemination and death. **(A)** Distribution of dissemination score according to anemia severity. **(B)** Distribution of deaths and time to death according to anemia severity. Early deaths were defined as deaths occurring within 7 days of enrolment, and late deaths were all deaths that occurred after 7 days and within 12 weeks of enrolment. **(C)** Hb levels according to dissemination score. **(D)** Hb levels according to time of death. Groups were compared using the Mann–Whitney U test. The Cochran–Armitage test for trend was used to assess the tendency of increased levels or frequencies among groups. **(E, F)** ROC curve analysis was used to evaluate the accuracy of Hb values to discriminate high dissemination score (Mtb dissemination score 3) **(E)** and early death **(F)**. Colored dots indicate the cut-off values of Hb extracted from the ROC curve analyses that resulted in the optimal ratio between sensitivity and specificity; these values are described in the indicated panels. Mild anemia was defined as Hb value >10 g/dL and <13 g/dL for men; and >10 and <12 g/dL for women, whereas moderate anemia was defined as Hb>8 g/dL and <=10 g/dL for both sexes. Severe anemia was defined as Hb<8g/dL for both sexes.

Next, we assessed the *Mtb* dissemination score and mortality according to the severity of anemia. Patients without anemia displayed a lower frequency of positive *Mtb* dissemination score (score 1, 2 and 3) than the other groups; 60.7% of patients without anemia had a *Mtb* dissemination score equal to zero (p<0.001). The frequency of participants with a positive *Mtb* dissemination score increased proportional to augmented anemia severity (p-value for trend <0.001) (**Figure 1A**). Mortality, including that occurring within the first 7 days of hospitalization (early death) tended to increase according to the severity of anemia (overall mortality: p-value for trend=0.009; early death: p-value for trend=0.018) (**Figure 1B**). When Hb values were examined as a continuous variable, we observed that its levels gradually decreased following increases in the *Mtb* dissemination score (p-value for trend <0.001) (**Figure 1C**). A correlation analysis confirmed those findings that the *Mtb* dissemination score and Hb values are inversely correlated (rho: -0.33; p<0.001). The opposite association was observed when comparing Hb levels with time of death, where those who had an early death presented the lowest Hb values compared with those who experienced late death (after 7 days of hospital admission) or survived (p-value for trend <0.001) (**Figure 1D**). Furthermore, mortality elevated following increases in the Mtb dissemination score (chi-square for trend p-value: <0.0001; **Supplementary Figure 3**).

A receiver operator characteristic (ROC) curve analysis was used to evaluate the accuracy of Hb levels for identifying persons with a high *Mtb* dissemination score (score = 3) (**Figure 1E**) or those who died early (**Figure 1F**). The results demonstrated that a Hb cut-off point of 8.05g/dL was associated with an overall accuracy of 65% (AUC: 0.65; 95%CI: 0.58-0.72), with a sensitivity of 58% (95%CI: 47-68) and specificity of 63% (95%CI: 58-68) (p<0.001) for identification of patients with high *Mtb* dissemination score (**Figure 1E**). Moreover, using a Hb cut-off value of 7.95g/dL resulted in an accuracy of 61% (AUC: 0.61, 95%CI: 0.51-0.72), with sensitivity of 66% (95%CI: 61-70) and specificity 60% (95%CI: 43-77, p=0.014) (**Figure 1F**).

### Severity of anemia is associated with the degree of inflammatory perturbation in HIV-associated TB

Lymphocyte (p<0.001) and monocyte (p<0.001) counts, as well as the concentrations of ALT (p=0.02) and albumin (p<0.001), decreased whereas values of CRP (p<0.001), procalcitonin (p<0.001), D-dimer (p<0.001), urea (p<0.001) and creatinine (p=0.018) showed a tendency to be higher according to anemia severity (**Supplementary Tables 2, 3**). Other comparisons between subgroups of individuals with different degrees of anemia are shown in **Supplementary Tables 2, 3**.

**Figure 2:**
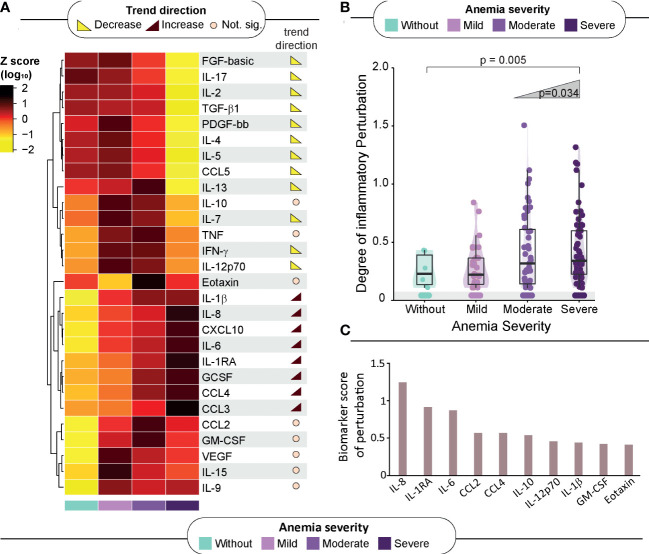
Association of anemia severity with inflammatory profile. **(A)** Left panel: A heatmap was designed to depict the overall pattern of inflammatory markers. A one-way hierarchical cluster analysis (Ward’s method) was performed. Dendrograms represent Euclidean distance. Right panel: Several analyses were performed to identify trends of increasing or decreasing of biomarker levels across anemia severity. Significant differences (p < 0.05) are highlighted in red-brown trend symbol when the trend is increasing and in yellow when the trend is decreasing. For those of no significance, there is a beige circle. **(B)** Scatter plots of the degree of inflammatory perturbation (DIP) value grouped according to anemia severity. Lines in the scatter plots represent median values and data were compared using the Mann–Whitney U test. The Cochran–Armitage test for trend was used to assess the tendency of increased levels or frequencies among groups. **(C)** We identified the Top 10 biomarker scores contributing to overall perturbation. The score was obtained using DIP approach. Mild anemia was defined as Hb value >10 g/dL and <13 g/dL for men; and >10 and <12 g/dL for women, whereas moderate anemia was defined as Hb>8 g/dL and <=10 g/dL for both sexes. Severe anemia was defined as Hb<8g/dL for both sexes.

The study groups were also compared according to the plasma concentrations of a variety of inflammatory markers to delineate the immunologic profile associated with anemia (**Figure 2A**). Trend analysis of IL-2, IL-5, FGF, PDGF-bb, IL-17, CCL5, IL-4, IL-13, IFN-γ, IL-7 and TGF-β1 uncovered that as the severity of anemia increased, the concentrations of these inflammatory molecules decreased. On the other hand, the circulating levels of IL-1β, IL-8, CXCL10, IL-6, CCL4, IL-1RA and CCL2 displayed an inverse behavior, with rising levels being proportional to increases in anemia severity (**Figure 2A**, right panel; **Supplementary Table 4**).

The abovementioned observations suggested that there is an intriguing disturbance of the immune activation systemically, which characterizes HIV-TB persons with severe anemia. To quantify such disturbance, we calculated the DIP scores in all the clinical groups, considering the non-anemic group as the reference group (**Figure 2B**). The resulting DIP scores were shown to inversely correlate with Hb values (rho: -0.22; p=0.007), and with CD4 cell counts (rho: -0.28; p=0.007) but were not related to HIV viral loads (**Supplementary Figures 4A–C**).

It was observed that the DIP score values increased following the severity of anemia (p-value for trend=0.005), reinforcing the idea that severe anemia in hospitalized patients with HIV-TB is associated with substantial inflammatory disturbance in the peripheral blood (**Figure 2B**). The top 10 biomarkers most contributing to this inflammatory perturbation (assessed through the DIP score) were IL-8, IL-1RA, IL-6, CCL4, CCL2, IL-10, IL-12p70, IL-1β, GM-CSF and Eotaxin (**Figure 2C**).

**Figure 3:**
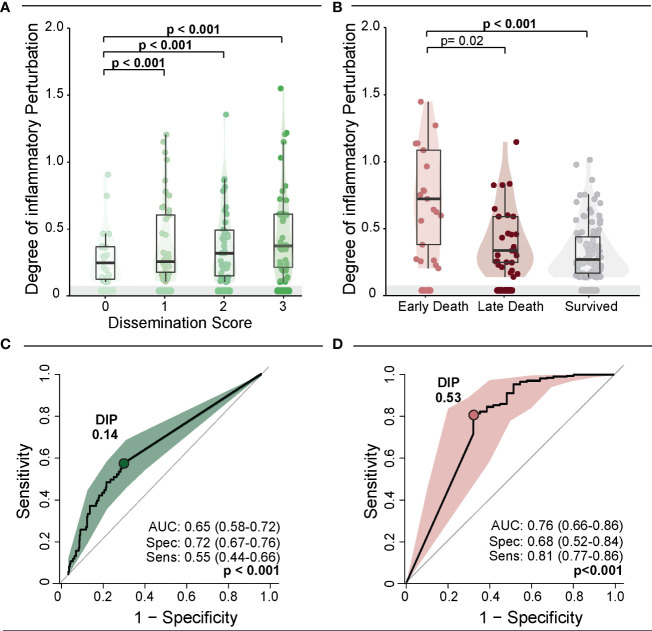
Degree of Inflammatory Perturbation (DIP) according to dissemination score and death. **(A)** Scatter plots of the DIP value grouped according to dissemination score. Lines in the scatter plots represent median values and data were compared using the Mann–Whitney *U* test. **(B)** Scatter plots of the DIP value grouped according to death. Lines in the scatter plots represent median values and data were compared using the Mann–Whitney *U* test. **(C, D)** ROC curve analysis was used to evaluate the accuracy of DIP values to discriminate high dissemination score (*Mtb* dissemination score 3) **(C)** and early death **(D)**. Colored dots indicate the cut-off values of DIP extracted from the ROC curve analyses that resulted in the optimal ratio between sensitivity and specificity; these values are described in the indicated panels.

We next designed analyses to test whether the DIP score values are somehow related to the degree of *Mtb* dissemination (**Figure 3A**). The DIP values gradually elevated proportional to increases of the *Mtb* dissemination score (p-value <0.001 for all the *ad hoc* comparisons). A correlation analysis confirmed that DIP values and Mtb dissemination score levels are directly related (rho: 0.31; p<0.001). This argues that systemic inflammatory profile is dramatically altered in patients that experience *Mtb* dissemination and that this association may be linked to anemia severity (**Figure 3A**). We next observed that DIP values were inversely correlated to the time to death (rho: -0.32; p=0.002). Indeed, patients who died within 7 days of hospital admission displayed substantially higher DIP score values than those who died at later timepoints or those who survived (p<0.001; **Figure 3B**).

Next, we used C-statistics analysis to evaluate the accuracy of the DIP score values identification of persons with a high *Mtb* dissemination score (score = 3) or of those who experienced early death. The accuracy for *Mtb* dissemination score using the DIP cut-off of 0.14 was 65% (AUC: 0.65; 95%CI: 58-72), with a sensitivity of 55% (95%CI: 0.44-0.66) and specificity of 72% (95%CI: 67-77) (p<0.001) (**Figure 3C**). The result of the ROC curve for early death was similar, with an accuracy of 76% (AUC:0.76; 95%CI: 0.66-0.86), with sensitivity of 81% (95%CI: 77-86) and specificity of 68% (95%CI: 52-84) (p<0.001) using a DIP cut-off point of 0.53 (**Figure 3D**). The shapes of the ROC curves indicate that DIP score values may be more suited for predicting early death than to estimate Mtb dissemination. Importantly, altogether, the results presented so far revealed that anemia (Hb values), systemic inflammation (DIP score values) and *Mtb* dissemination are all interrelated and impact overall mortality and time to death.

**Figure 4:**
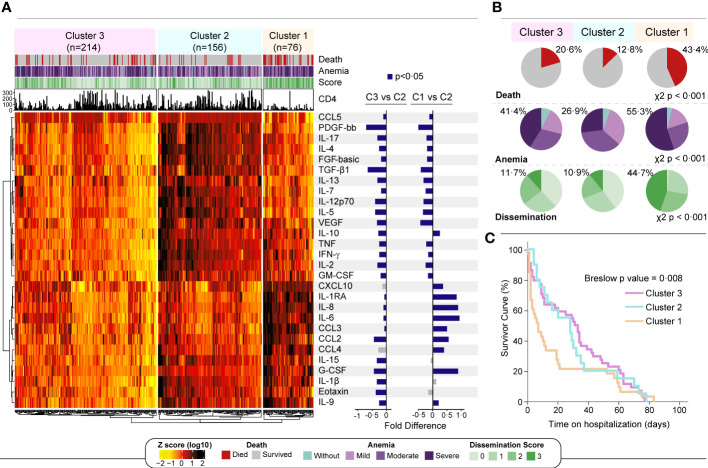
A more pronounced inflammatory profile is associated with severe anemia and death. **(A)** Left panel: An unsupervised two-way hierarchical cluster (Ward’s method) was performed with all 496 participants. Data were log10 transformed and ranked and colored in a heatmap from values detected for each inflammatory biomarker. Dendrograms represent Euclidean distance. Three main clusters were defined. Right panel: A log10 fold change was performed comparing each cluster with reference cluster (C2, due to lowest frequency of dissemination and death). Significant differences (p < 0.05) are highlighted in blue bars. **(B)** For each cluster, shaded areas represent frequency of death (red), anemia severity grade (light blue to dark purple) and *Mtb* dissemination score (light green to dark green) and. Chi squared test was performed to each variable comparing clusters. **(C)** Survivor curves show the probability of survival over 12 weeks for each cluster. Mild anemia was defined as Hb value >10 g/dL and <13 g/dL for men; and >10 and <12 g/dL for women, whereas moderate anemia was defined as Hb>8 g/dL and <=10 g/dL for both sexes. Severe anemia was defined as Hb<8g/dL for both sexes.

Finally, an unsupervised hierarchical cluster analysis was performed using biomarker measurements from all study participants to examine whether there is a unique profile that characterizes those who die faster. Three main clusters of patients were defined (**Figure 4A**). The cluster #1 displayed a higher frequency of participants who died during the follow up and had the uppermost occurrence of patients with high *Mtb* dissemination scores and severe anemia (p<0.001 in both comparisons) (**Figure 4B**). The cluster #2 exhibited a lower frequency of anemic participants, lower frequency of people with any *Mtb* dissemination and lower mortality than the other clusters/sub-groups and was considered as the reference for fold difference comparisons (**Figure 4A**, right panel). The cluster #3 included participants with an intermediate phenotype, without any characteristics that specifically defined this group (**Figure 4B**). When we compared those three clusters, the individuals within the cluster #1 presented relatively higher values of cytokines and chemokines, such as CXCL10, IL-1RA, IL-6, IL-8, CLL2, CCL3 and CCL4 than those in the other clusters (**Supplementary Table 5**). This finding shows once again that these inflammatory mediators are involved with the inflammatory exacerbation in patients with severe anemia and that this setting is related with *Mtb* dissemination and death in the study population. Finally, the survival analysis demonstrated that patients from cluster #1 had higher mortality than those from the other clusters (p=0.008) (**Figure 4C**).

## Discussion

Anemia affects one third of the world’s population, and mainly in PLHIV and in those with TB (22, 23). In a previous study from our group examining persons with HIV-TB co-infection, anemia was reported in 84% of the participants (20). In addition, in many reports, the majority of the participants examined present with mild anemia (2, 24). The present cohort study revealed that 92.7% of study participants had anemia, with a high proportion of severe anemia (38%). This higher frequency may be explained by the fact that we enrolled only hospitalized patients. Yet, regardless of its imposing pervasiveness in such setting, anemia is frequently overlooked in the clinical practice when patients with HIV-TB are managed. No consensus on how anemia in HIV-TB patients should be addressed has been documented. The findings presented here demonstrate that Hb levels not only infer TB dissemination but also indicate degree of inflammatory disturbance. More importantly, Hb levels are predictive of early mortality in hospitalized persons with HIV-associated TB. Whether anemia is a cause or a consequence of the HIV-TB-driven chronic inflammation and/or disease progression is less important than its utility as a biomarker that can identify persons at higher risk of death. Early identification of such patients through a simple measurement of Hb levels must alarm the healthcare professionals to take a closer look and optimize management to reduce the odds of mortality. Future investigations are warranted to test whether early interventions, such as use of adjunct therapies, fostered by Hb measurement at hospital admission impact survival of this vulnerable population.

In this cohort, most of the participants with moderate to severe anemia were women. This can be explained by different reasons, such as heavy menstrual bleeding and uterine conditions that may be associated with blood loss. In addition, it is already known that women have a higher frequency of anemia than men, regardless of the primary cause of anemia (25). In addition, anemic patients were shown to have a lower weight and lower CD4 count, while having higher frequency of positive urine *Mtb* Xpert test and detectable CMV in blood. Other studies have demonstrated that anemic patients frequently have weight loss and lower CD4 counts (5), which supports the conclusion that anemia is related to more advanced forms of TB and HIV. The association between low Hb values with low CD4 T lymphocyte count and high frequency of CMV co-infection suggests that anemia can be caused by advanced HIV with opportunistic infections such as TB and/or CMV. Decreased levels of Hb have been described as predictive markers for HIV disease progression to AIDS (26). We found that Hb levels were just weakly positively correlated with CD4 cell counts, and not related with HIV viral loads. At first glance, this observation may indicate that the relationship between anemia and HIV progression was not substantially strong in our cohort. That could be due to the fact that we mostly enrolled persons with already advanced disease (median CD4 count: 56 cells/µL). Therefore, we are unable to test the association between Hb values and HIV disease progression in a proper study design.

Our multidimensional analyses exploring the relationships between Hb values and TB progression confirmed the previously established hypothesis that anemia hallmarks advanced TB disease. The data on *Mtb* dissemination score reported here highlight that persons with severe anemia are those presenting with more frequent detection of *Mtb* in extrapulmonary compartments such as urine and blood. *Mtb* dissemination is reported to occur when growth of mycobacteria is unfettered, which is observed when the infected host is unable to adequately respond with a robust and efficient immune response (reviewed in [27)]. Such incapacity to defeat *Mtb* is frequently observed in immunocompromised persons, that include those living with HIV. When we combine our results on relationships between Hb values and surrogates of HIV (CD4 count and HIV viral load) or of TB (dissemination score), we can argue that anemia in this study population is likely more noticeably related to progression of TB progression than that driven by HIV. Corroborating with this idea, a retrospective cohort study of persons with advanced HIV (CD4 <100 cells/µL) reported that those with TB diagnosis more frequently had anemia and exhibited more pronounced inflammatory profile than those without this comorbidity (20).

An important criticism commonly emerges during discussion of results such as those reported here. That is related with the difficulty to establish whether anemia is an underlying factor driving the inflammatory abnormalities or is a consequence of sustained immunopathology. In fact, our analysis demonstrated that there is a strong relationship between the severity of anemia and the degree of systemic inflammatory perturbation. The study design does not allow us to determine causality. Instead, it sanctions the use of Hb levels as a proxy of inflammatory disturbance and of a unique immune activation profile that relates with *Mtb* dissemination and mortality. Thus, Hb is a simple, low-cost parameter that deserves more attention, especially in limited-resource regions.

The unique inflammatory profile observed in patients with severe anemia who experienced TB dissemination includes high concentrations of IL-1RA, IL-8 and IL-6, all of which have been previously described to be involved in mycobacteria-associated immunopathology in both clinical and experimental settings (28–33). Moreover, these heightened levels of these cytokines have been previously reported as risk factors of TB progression and infer increased morbidity and mortality (34–36). Curiously, these cytokines are closely associated with innate immune responses, and cells described to rapidly respond to its induced signals are macrophages and neutrophils, which have been placed as critical cells driving both immunity against TB (37, 38) and immune-driven tissue damage (39, 40). The predominance of signals deriving from activation of innate immune responses over the molecules fostering T cell activity favors the hypothesis that innate cells may play a more substantial role in induction of the systemic inflammatory perturbation reported here. Whether these cytokines are insufficiently attempting to control TB dissemination or are solely promoting immunopathology that is leading to worse outcomes is still a matter of debate and deserves additional investigations.

Two cytokines deserve extended discussion, due to its well-described associations with both TB disease and anemia. IL-6 is a multifunctional cytokine that induces the production of hepcidin, which inhibits iron absorption, blocking the release of iron from macrophages and the delivery of heme to erythroid cells (41). The involvement of IL-6 in the production of hepcidin contributes to the development and aggravation of anemia of inflammation. Increases in hepcidin (reflecting in increases in IL-6 concentrations) has already been described as a predictor of mortality, as well as incident TB and increased *Mtb* dissemination in PLHIV (18, 42). IL-8 is an important chemoattractant and cell activator, that plays an important role in the pathogenesis of HIV-TB (43). IL-8 recruits immune cells to the site of infection/inflammation and its high concentrations have been described as risk factors for death from TB and sepsis (28). Furthermore, high concentrations of IL-8 have also been described in PLHIV with mild anemia at baseline whose anemia does not recover even after starting antiretroviral treatment (44). Importantly, both IL-6 and IL-8 were among the markers found to be driving the overall DIP score values in our study. Combined, these immune mediators may be accounting for the effects on the overall inflammatory disturbance which is either driving or being driven by anemia.

The worse possible outcome that can occur in patients severely afflicted by TB and HIV infections is death. In our cohort, 22% of the participants died, and 7% died within a week of admission. An omnipresent feature of the patients who died seemed to be the presence of anemia, given that only 5 (5%) participants who died at any timepoint were not anemic at baseline. Thus, Hb levels are a strong predictor of mortality in persons with HIV-associated TB. This important observation is supported by a systematic review which demonstrated that anemia, regardless of its type, is associated with an increased risk of all-cause mortality in PLHIV (12). Discriminant analysis using C-statistics demonstrated that a Hb value <8 g/dL can identify patients with high *Mtb* dissemination score (score=3). Similarly, baseline Hb values can reliably predict early mortality (AUC: 0.63, p=0.008), which is higher than that of other parsimonious biomarkers previously described, such as CRP (AUC: 0.31, p=1.0) and D-dimer (AUC: 0.30, p=0.06). If validated in other studies, assessment of Hb should be not ignored as an important predictor which may drive change/optimization of clinical management.

This study has certain limitations. Samples were obtained only at baseline, not allowing the evaluation of changes during time of treatment. Additionally, we did not have access to data on confirmed cause of death, including autopsy information. A distinct study is currently retrieving such information. Regardless, our study identifies previously underexplored nuances of the key relationships between anemia, inflammation, and control of pathogen loads/dissemination in highly susceptible patients with HIV-associated TB. More importantly, the findings propose the systematic implementation of Hb measurement as a mandatory policy that may reduce the extremely high mortality in this population.

## Data availability statement

The raw data supporting the conclusions of this article will be made available by the authors, without undue reservation.

## Ethics statement

The studies involving human participants were reviewed and approved by University of Cape Town Human Research Ethics Committee (UCT HREC), reference number 057/2013. The patients/participants provided their written informed consent to participate in this study.

## Author contributions

Conceptualization, MA-P, CS, GM and BA; Data curation, CS, DB, AW; Investigation, MA-P, CS, GM and BA; Formal analysis, MA-P, CS, DB and BA; Methodology, MA-P, and BA; Software, MA-P; Supervision, GM, and BA; Writing—original draft, MA-P, CS, BB-D, DB, KV-S, CV, GM and BA; Writing—review and editing, all authors. All authors contributed to the article and approved the submitted version.
