# Supplementary material for: Interplay between systemic inflammation, anemia, and mycobacterial dissemination and its impact on mortality in TB-associated HIV: a prospective cohort study
Source: Front Immunol. 2023 Apr 18;14:1177432. doi: 10.3389/fimmu.2023.1177432 (PMC10151654; doi:10.3389/fimmu.2023.1177432)
Supplement: Supplementary file 1 [file DataSheet_1.pdf]

Supplementary Material

SUPPLEMENTARY METHODS

Exclusion criteria

Pregnant women, history of anti-TB therapy within the last month, or those who were recently initiated and received three or more doses of anti-TB therapy were not eligible for enrolment.

Ethics Statement

Eligible patients with a decreased level of consciousness were enrolled and followed up daily until they regained the capacity to participate in the informed consent process, and if not agreeable to participate, were withdrawn from the study. The UCT HREC approved the use of information from participants who died prior to providing informed consent by the end of the study follow-up.

Laboratory assays

Plasma was stored at −80°C for immunology assays. Soluble inflammatory mediators were tested on stored plasma (1:2 dilution) using Luminex technology (Bio-Plex Pro Human Cytokine Standard 27-Plex kit). The following analytes were measured: interleukin (IL)-1β, IL-1 receptor antagonist (IL-1Ra), IL-2, IL-4, IL-5, IL-6, IL-7, IL-8, IL-9, IL-10, IL-12p70, IL-13, IL-15, IL-17A, eotaxin, basic fibroblast growth factor (FGF), granulocyte colony stimulating factor (G-CSF)/colony stimulating factor 3 (CSF3), granulocyte-macrophage colony stimulating factor (GM-CSF/CSF2), interferon gamma (IFN-γ), interferon gamma-induced protein (IP-10)/ C-X-C motif chemokine ligand 10 (CXCL10), monocyte chemoattractant protein-1 (MCP-1)/C-C motif chemokine ligand 2 (CCL2), macrophage inflammatory protein-1 alpha (MIP-1α/CCL3), MIP-1 beta (MIP-1β/CCL4), platelet-derived growth factor-BB (PDGF), regulated on activation, normal T cell expressed and secreted (RANTES/CCL5), tumor necrosis factor-alpha (TNF), and vascular endothelial growth factor (VEGF). For statistical analyses, mean fluorescence intensity (MFI) values of the plasma markers were used. Such approach allows for analysis of analytes of low abundance and does not require censoring or correction for background(Breen et al., 2016, 2015; Schutz et al., 2019).

Degree of Inflammatory Perturbation

The degree of inflammatory perturbation (DIP) was calculated to identify the general inflammatory environment of the participants. DIP was adapted from the molecular degree of perturbation, which has been described previously(Gonçalves et al., 2019). For this study, the DIP calculation included the concentrations of the plasma inflammatory markers instead of gene expression values in the original analysis model(Gonçalves et al., 2019). Thus, herein, the average level and standard deviation of a baseline reference group (without anemia) were calculated for each biomarker. The DIP score of each biomarker was defined by z-score normalization, where the differences in concentration values from the average of the biomarker in reference group was divided by the reference standard deviation. Therefore, the DIP score represents the differences by number of standard deviations from the control group. Similar approaches resulting in DIP-like scores have been previously employed using biomarker measurements by our group(Demitto et al., 2020; Oliveira-de-Souza et al., 2019). We ranked the top

10 markers which contributed the most for the DIP score values, to identify the most informative soluble mediators contributing to the overall inflammatory disturbance.

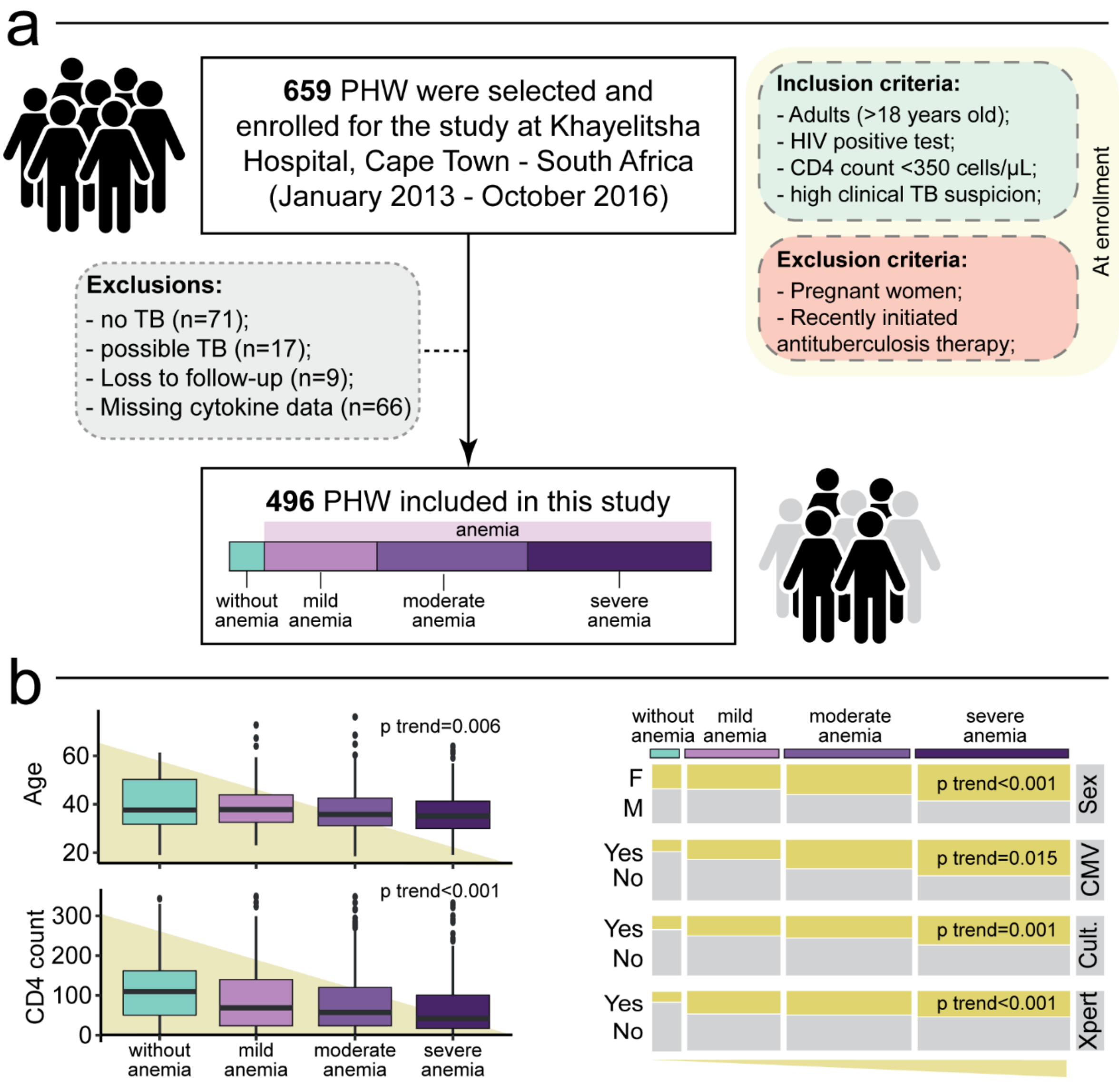

Supplementary Fig 1. Study Flow chart. (a) 659 People with HIV (PWH) were selected and enrolled for the study, however, 193 were removed for this analysis, leaving 496 patients. Of these, the majority (92.7%) were anemic with different severities. (b) Clinical characteristics of patients compared according to anemia severity. Left panel: Decreasing trends in age and CD4 counts were observed with increasing severity of anemia. The trend direction is represented by the colored shaded triangle. On the other hand, there were also trends towards an increase in frequency (shown as %) of the female sex, CMV viremia, positive *Mtb* blood culture, and positive Urine Xpert as the severity of anemia increased (right panel). The Cochran–Armitage test for trend was used to assess the tendency of increased levels or frequencies among groups. Mild anemia was defined as Hb value >10 g/dL and <13 g/dL for men; and >10 and <12 g/dL for women, whereas moderate anemia was defined as Hb>8 g/dL and <=10 g/dL for both sexes. Severe anemia was defined as Hb<8g/dL for both sexes.

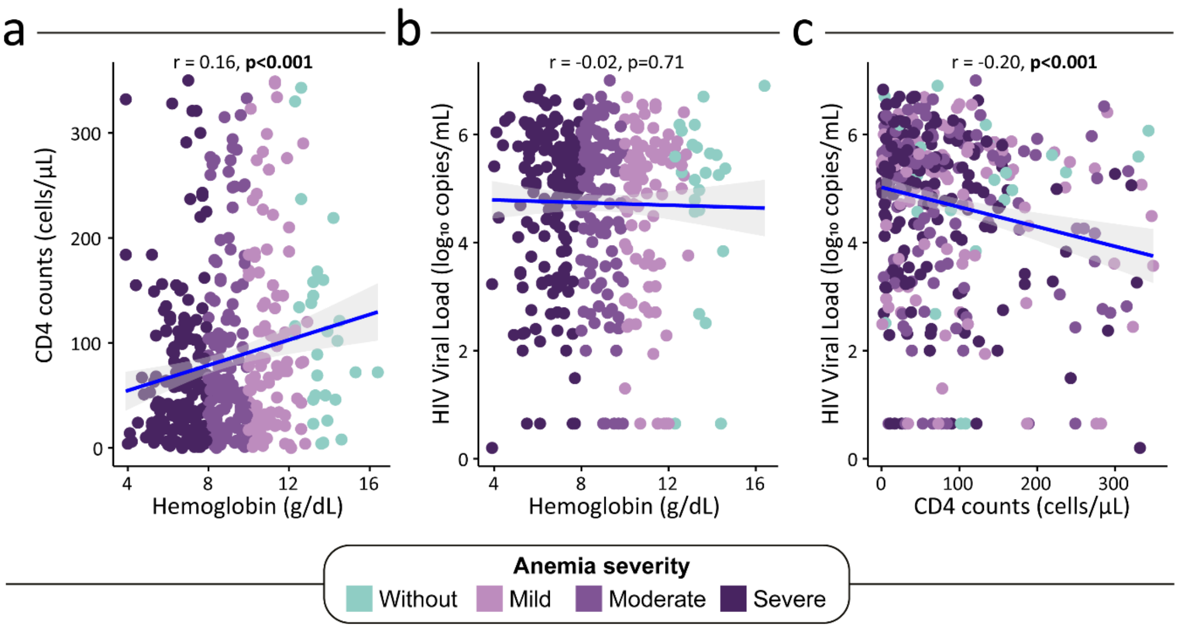

Supplementary Fig 2. Spearman correlation analysis between hemoglobin values, CD4 counts, and log10 HIV viral load. (a) Spearman correlation between CD4 counts vs hemoglobin. (b) Spearman correlation between log10 HIV viral load vs hemoglobin. (C) Spearman correlation between log10 HIV viral load vs CD4 count. (Mild anemia was defined as Hb value >10 g/dL and <13 g/dL for men; and >10 and <12 g/dL for women, whereas moderate anemia was defined as Hb>8 g/dL and <=10 g/dL for both sexes. Severe anemia was defined as Hb<8g/dL for both sexes.

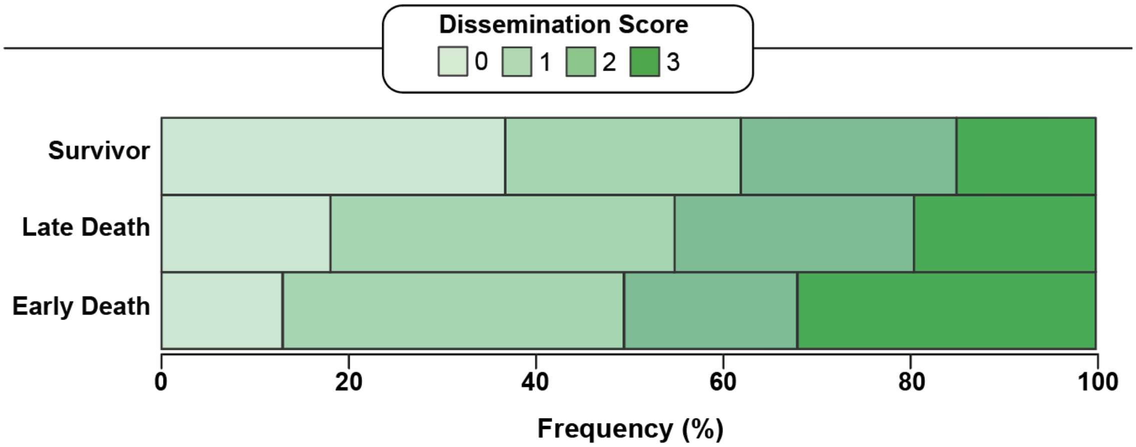

	Dissemination Score 0	Dissemination Score 1	Dissemination Score 2	Dissemination Score 3
Survivor	36.7%	25.2%	22.9%	15.2%
Late Death	18.2%	36.4%	25.8%	19.7%
Early Death	12.9%	35.5%	19.4%	32.3%

Supplementary Fig 3. Frequency of *Mtb* dissemination score according to time of death. Upper panel: The frequency (%) of *Mtb* dissemination score is shown in a bar graph, with groups stratified by time of death (early, late, and survivor). Down panel: The frequency (%) of *Mtb* dissemination score is shown in a table format, with groups stratified by time of death (early, late, and survivor). Early death was defined as death that occurred in the first seven days after hospitalization. Groups were compared using the chi-square test and chi-square for trend.

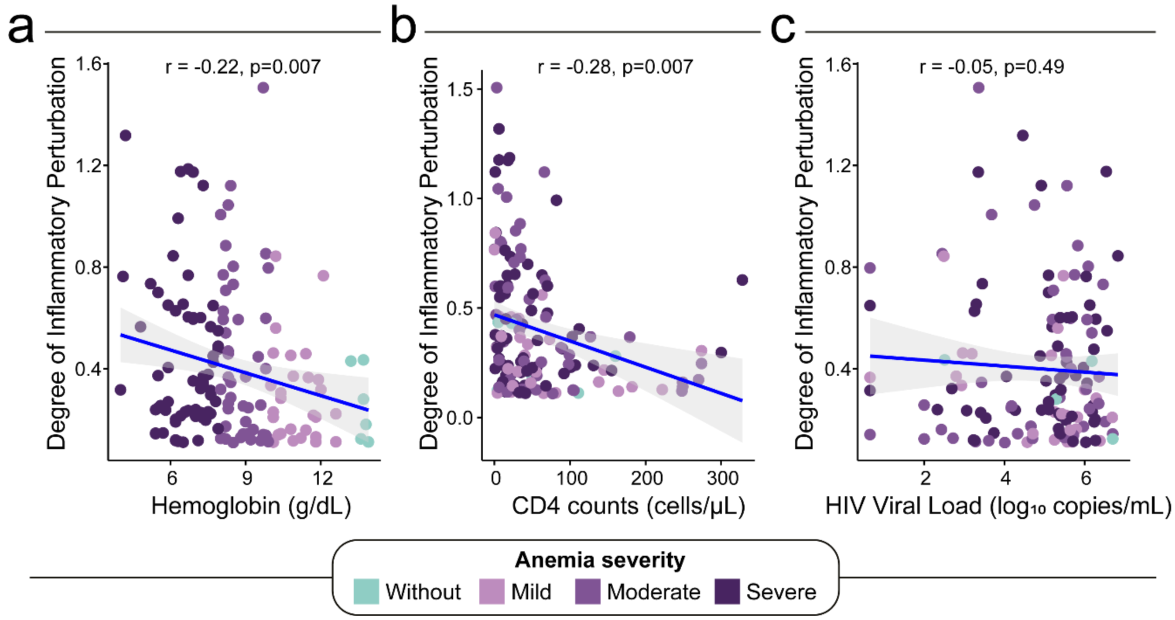

Supplementary Fig 4. Spearman correlation analysis between Degree of Inflammatory Perturbation (DIP), hemoglobin values, CD4 counts, and log10 HIV viral load. (a) Spearman correlation between DIP vs hemoglobin. (b) Spearman correlation between DIP vs CD4 counts. (c) Spearman correlation between DIP vs log10 HIV viral load. Mild anemia was defined as Hb value >10 g/dL and <13 g/dL for men; and >10 and <12 g/dL for women, whereas moderate anemia was defined as Hb>8 g/dL and <=10 g/dL for both sexes. Severe anemia was defined as Hb<8g/dL for both sexes.

Definition	Hb Level (g/dL)
Anemia	<13 for men<br><12 for women
Mild Anemia	10 ≤ <13 for men<br>10 ≤ <12 for women
Moderate anemia	8 ≤ <10 for men<br>8 ≤ <10 for women
Severe Anemia	<8 for both sexes

Supplementary Fig 5. Anemia definition according to the WHO Criteria, using hemoglobin levels (World Health Organization, 2011).

Supplementary Table 1. Clinical characteristics according to the presence of anemia

	All (n=496)	Without anemia (n=36)	With anemia (n=460)	p value
Sex (female), n (%):	262 (52.8)	15 (41.7)	247 (53.7)	0.223
Age (years), median (IQR):	35.9 (30.9-43.2)	37.6 (31.8-50.2)	35.9 (30.9-42.8)	0.306
Weight (kg), median (IQR):	54.0 (47.0-62.0)	57.0 (49.2-75.8)	54.0 (47.0-61.0)	**0.025**
ART naive, n (%):	198 (40.0)	19 (52.8)	179 (39.0)	0.256
CD4 (count), median (IQR):	57.0 (21.0-117)	110 (50.0-162)	55.0 (20.0-111)	**0.001**
HIV VL (log_{10} copies/mL), median (IQR)	5.22 (3.83-5.75)	5.25 (4.35-5.70)	5.21 (3.75-5.75)	0.809
CMV detected, n (%):	192 (39.2)	6 (16.7)	186 (41.0)	**0.007**
MTB blood culture, n (%):	188 (39.4)	7 (22.6)	181 (40.6)	0.139
Urine Xpert positive, n (%):	199 (46.4)	6 (18.8)	193 (48.6)	**0.002**

Table note:

Bold font indicates statistical significance. Data are shown as median and interquartile (IQR) range or frequency (percentage). Categorical data were compared between the clinical groups using the Chi-squared tests. Continuous data were compared between the clinical groups using the Mann-Whitney *U* test (for two unmatched groups). Mild anemia was defined as Hb value >10 g/dL and <13 g/dL for men; and >10 and <12 g/dL for women, whereas moderate anemia was defined as Hb>8 g/dL and <=10 g/dL for both sexes. Severe anemia was defined as Hb<8g/dL for both sexes. Abbreviations: IQR: Interquartile range; ART: Antiretroviral treatment; VL: viral load; CMV: cytomegalovirus; MTB: *Mycobacterium tuberculosis;*

Supplementary Table 2. Cellular and biochemical profile according anemia severity

	Without anemia (n=36)	Mild anemia (n=116)	Moderate anemia (n=155)	Severe anemia (n=189)	p value	p trend
Hemoglobin (g/dL), median (IQR):	13.4 (12.9-14.2)	10.8 (10.4-11.4)	8.80 (8.30-9.35)	6.80 (6.00-7.30)	**<0.001**	**<0.001**
Mean Corpuscular Volume (fl)	85.2 (81.4-90.3)	85.1 (81.2-89.1)	81.2 (77.5-86.0)	79.1 (74.0-84.9)	**<0.001**	**<0.001**
White cell count ($\times10^{9}$/L), median (IQR)	6.42 (4.76-9.40)	6.96 (4.86-9.88)	7.15 (4.41-9.80)	7.25 (4.07-11.7)	0.824	0.896
Abs. lymphocyte count ($\times10^{9}$/L), median (IQR)	1.02 (0.58-1.22)	0.63 (0.38-1.15)	0.56 (0.33-0.85)	0.50 (0.29-0.82)	**<0.001**	**<0.001**
Abs. monocyte count ($\times10^{9}$/L), median (IQR)	0.45 (0.19-0.66)	0.42 (0.24-0.64)	0.31 (0.15-0.51)	0.28 (0.13-0.56)	**0.001**	**0.001**
Abs. neutrophil count ($\times10^{9}$/L), median (IQR)	5.11 (3.42-7.26)	5.40 (3.34-8.12)	5.84 (3.14-8.63)	5.72 (3.21-8.88)	0.831	0.414
Platelet count ($\times10^{9}$/L), median (IQR)	258 (190-297)	279 (187-362)	272 (174-362)	252 (163-332)	0.148	0.124

Table note:

Bold font indicates statistical significance. Mild anemia was defined as Hb value >10 g/dL and <13 g/dL for men; and >10 and <12 g/dL for women, whereas moderate anemia was defined as Hb>8 g/dL and <=10 g/dL for both sexes. Severe anemia was defined as Hb<8g/dL for both sexes. Data are shown as median and interquartile (IQR) range or frequency (percentage). Categorical data were compared between the clinical groups using the Chi-squared tests. Continuous data were compared between the clinical groups using the Kruskall-Wallis test. [a]without anemia x mild anemia; [b]without anemia x moderate anemia; [c]without anemia x severe anemia; [d]mild x moderate; [e]mild x severe; [f]moderate x severe. Abbreviations: IQR: Interquartile range.

Supplementary Table 3. Cellular and biochemical profile according anemia severity

	Without anemia (n=36)	Mild anemia (n=116)	Moderate anemia (n=155)	Severe anemia (n=189)	p value	p trend
Random glucose (mmol/L), median (IQR)	5.30 (4.68-5.93)	5.20 (4.60-6.00)	5.20 (4.80-6.00)	5.30 (4.70-6.20)	0.746	0.553
Venous lactate (mmol/L), median (IQR)	1.45 (1.20-2.10)	1.70 (1.28-2.10)	1.80 (1.30-2.75)	2.00 (1.40-2.70)	0.06	**0.008**
C-reactive protein (mg/L), median (IQR)	77.8 (44.8-155)	148 (75.1-217)	146 (96.4-228)	175 (113-234)	**<0.001**	**<0.001**
Procalcitonin (µg/L), median (IQR)	0.30 (0.08-1.97)	0.72 (0.21-4.67)	2.04 (0.34-6.13)	4.74 (1.52-19.0)	**<0.001**	**<0.001**
D-dimer (mg/L), median (IQR)	0.66 (0.40-1.39)	1.19 (0.84-2.70)	1.27 (0.97-3.63)	2.35 (1.10-4.12)	**<0.001**	**<0.001**
AST (U/L), median (IQR)	45.5 (29.5-85.2)	52.0 (31.0-92.5)	57.0 (34.0-118)	57.0 (36.0-92.0)	0.43	0.26
ALT (U/L), median (IQR)	33.0 (22.5-57.0)	29.0 (17.0-50.0)	30.5 (16.0-54.0)	23.0 (15.0-38.0)	**0.002**	**0.002**
GGT (U/L), median (IQR)	74.0 (41.0-122)	75.0 (37.5-182)	85.5 (49.2-161)	73.0 (42.0-138)	0.454	0.867
Alkaline phosphatase (U/L), median (IQR)	101 (63.2-172)	106 (79.2-148)	122 (83.0-192)	118 (73.0-179)	0.141	0.299
Total bilirubin (µmol/L), median (IQR)	7.50 (5.00-13.0)	8.00 (5.00-11.8)	8.00 (6.00-12.0)	7.00 (5.00-12.0)	0.776	0.787
Conjugated bilirubin (µmol/L), median (IQR)	4.00 (2.00-7.75)	4.00 (2.00-6.00)	4.00 (3.00-7.50)	4.00 (3.00-8.00)	0.502	0.242
Total protein (g/L), median (IQR)	78.5 (73.0-87.0)	76.0 (70.0-84.0)	76.0 (67.0-85.0)	72.0 (65.2-81.0)	**0.002**	**<0.001**
Albumin (g/L), median (IQR)	33.5 (28.0-39.0)	27.0 (23.0-30.0)	25.0 (21.0-28.0)	23.0 (19.0-26.0)	**<0.001**	**<0.001**
Sodium (mEq/L), median (IQR)	130 (126-133)	129 (125-132)	128 (124-131)	128 (125-131)	0.357	0.15
Potassium (mmol/L), median (IQR)	4.20 (3.85-4.65)	4.10 (3.60-4.55)	3.90 (3.50-4.60)	3.90 (3.40-4.43)	0.149	0.022
Urea (mg/dL), median (IQR)	4.10 (2.88-7.53)	4.95 (3.42-8.97)	5.00 (3.45-8.60)	6.50 (4.00-11.4)	**0.003**	**<0.001**
Creatinine (µmol/L), median (IQR)	75.0 (62.2-97.0)	79.5 (61.0-111)	77.0 (58.0-116)	89.0 (63.0-158)	0.06	**0.018**

Table note:

Bold font indicates statistical significance. Mild anemia was defined as Hb value >10 g/dL and <13 g/dL for men; and >10 and <12 g/dL for women, whereas moderate anemia was defined as Hb>8 g/dL and <=10 g/dL for both sexes. Severe anemia was defined as Hb<8g/dL for both sexes. Data are shown as median and interquartile (IQR) range or frequency (percentage). Categorical data were compared between the clinical groups using the Chi-squared tests. Continuous data were compared between the clinical groups using the Kruskall-Wallis test. [a]without anemia x mild anemia; [b]without anemia x moderate anemia; [c]without anemia x severe anemia; [d]mild x moderate; [e]mild x severe; [f]moderate x severe. Abbreviations: IQR: Interquartile range.

Supplementary Table 4. Inflammatory profile according to anemia severity

	Without anemia (n=36)	Mild anemia (n=116)	Moderate anemia (n=155)	Severe anemia (n=189)	p-value	p trend
**IL-1β**	1.72 (1.57-1.86)	1.79 (1.63-1.89)	1.83 (1.71-1.94)	1.83 (1.72-1.97)	**0.001**	**<0.001**
**IL-1RA**	1.99 (1.85-2.31)	2.10 (1.94-2.47)	2.31 (1.99-2.71)	2.46 (2.14-2.88)	**<0.001**	**<0.001**
**IL-2**	1.83 (1.72-1.92)	1.83 (1.75-1.91)	1.83 (1.75-1.92)	1.79 (1.71-1.88)	0.057	**0.02**
**IL-4**	1.69 (1.53-1.80)	1.71 (1.59-1.84)	1.68 (1.57-1.80)	1.61 (1.46-1.75)	**<0.001**	**<0.001**
**IL-5**	1.50 (1.36-1.66)	1.52 (1.36-1.69)	1.48 (1.34-1.65)	1.38 (1.23-1.53)	**<0.001**	**<0.001**
**IL-6**	2.04 (1.77-2.28)	2.25 (2.04-2.51)	2.35 (2.10-2.67)	2.45 (2.25-2.72)	**<0.001**	**<0.001**
**IL-7**	1.51 (1.45-1.62)	1.57 (1.45-1.68)	1.56 (1.44-1.67)	1.49 (1.38-1.59)	**0.002**	**0.002**
**IL-8**	1.87 (1.79-2.09)	1.99 (1.84-2.19)	2.06 (1.92-2.28)	2.18 (2.00-2.46)	**<0.001**	**<0.001**
**IL-9**	2.18 (2.02-2.32)	2.20 (2.08-2.31)	2.19 (2.08-2.33)	2.19 (2.08-2.32)	0.713	0.498
**IL-10**	1.82 (1.72-1.89)	1.86 (1.73-1.97)	1.85 (1.76-1.95)	1.82 (1.73-1.91)	0.061	0.078
**IL-12p70**	1.69 (1.61-1.87)	1.77 (1.64-1.89)	1.76 (1.62-1.91)	1.69 (1.59-1.82)	**0.007**	0.**009**
**IL-13**	1.57 (1.46-1.72)	1.59 (1.48-1.76)	1.61 (1.47-1.85)	1.51 (1.34-1.69)	**<0.001**	**<0.001**
**IL-15**	1.93 (1.86-2.06)	1.97 (1.87-2.07)	1.96 (1.88-2.06)	1.95 (1.86-2.05)	0.544	0.528
**IL-17**	1.87 (1.64-1.98)	1.86 (1.71-2.02)	1.81 (1.71-1.97)	1.73 (1.62-1.87)	**<0.001**	**<0.001**
**Eotaxin**	1.81 (1.66-1.89)	1.80 (1.70-1.90)	1.83 (1.71-1.97)	1.81 (1.70-1.96)	0.227	0.159
**FGF**	1.75 (1.62-1.86)	1.76 (1.65-1.87)	1.72 (1.64-1.83)	1.68 (1.59-1.77)	**<0.001**	**<0.001**
**GCSF**	1.79 (1.66-1.88)	1.80 (1.69-1.91)	1.85 (1.74-1.99)	1.86 (1.73-2.04)	**0.012**	**0.002**
**GMCSF**	1.90 (1.86-2.01)	1.94 (1.85-2.07)	1.96 (1.87-2.06)	1.93 (1.82-2.04)	0.148	0.706
**IFN-γ**	1.66 (1.51-1.93)	1.75 (1.59-1.88)	1.74 (1.61-1.88)	1.67 (1.54-1.83)	**0.006**	**0.017**
**CXCL10**	3.48 (3.08-3.78)	3.76 (3.42-4.07)	3.85 (3.56-4.10)	3.99 (3.76-4.16)	**<0.001**	**<0.001**
**CCL4**	1.95 (1.79-2.05)	1.97 (1.87-2.12)	2.00 (1.89-2.15)	2.01 (1.88-2.20)	**0.019**	**0.003**
**CCL2**	1.86 (1.73-2.14)	1.96 (1.80-2.17)	2.03 (1.84-2.25)	1.97 (1.83-2.28)	**0.049**	0.106
**PDGF-bb**	2.30 (1.97-2.67)	2.40 (1.98-2.72)	2.27 (1.91-2.56)	2.06 (1.80-2.41)	**<0.001**	**<0.001**
**CCL3**	2.74 (2.55-3.02)	2.76 (2.61-2.99)	2.83 (2.63-3.04)	2.91 (2.68-3.17)	**0.001**	**<0.001**
**CCL5**	4.19 (4.14-4.23)	4.20 (4.12-4.22)	4.18 (4.11-4.22)	4.14 (3.98-4.20)	**<0.001**	**<0.001**
**TNF**	1.60 (1.52-1.69)	1.65 (1.57-1.73)	1.65 (1.57-1.75)	1.61 (1.51-1.72)	**0.028**	**0.14**
**VEGF**	1.96 (1.81-2.14)	2.05 (1.87-2.23)	2.03 (1.91-2.21)	2.01 (1.88-2.16)	0.341	0.513
**TGF-β1**	1.49 (1.19-1.71)	1.49 (1.19-1.78)	1.47 (1.19-1.75)	1.25 (1.10-1.56)	**<0.001**	**<0.001**

Table note:

Bold font indicates statistical significance. Mild anemia was defined as Hb value >10 g/dL and <13 g/dL for men; and >10 and <12 g/dL for women, whereas moderate anemia was defined as Hb>8 g/dL and <=10 g/dL for both sexes. Severe anemia was defined as Hb<8g/dL for both sexes. Data are shown as median and interquartile range (IQR). The Kruskall-Wallis test was used to assess the statistical differences between all

groups. The Cochran–Armitage test for trend was used to assess for the presence of an association between the measurements and the severity of anemia. Abbreviations: IQR: Interquartile range.

Supplementary Table 5. Clinical and Inflammatory profile according to cluster

	Cluster 3 (n=214)	Cluster 2 (n=156)	Cluster 1 (n=76)	P value
**Anemia severity, n (%)**				**<0.001**
**Without**	16 (7.48)	9 (5.77)	3 (3.95)	
**Mild**	46 (21.5)	48 (30.8)	12 (15.8)	
**Moderate**	64 (29.9)	57 (36.5)	19 (25.0)	
**Severe**	88 (41.1)	42 (26.9)	42 (55.3)	
**Dissemination score, n (%)**				**<0.001**
**0**	81 (37.9)	62 (39.7)	1 (1.32)	
**1**	57 (26.6)	46 (29.5)	20 (26.3)	
**2**	51 (23.8)	31 (19.9)	21 (27.6)	
**3**	25 (11.7)	17 (10.9)	34 (44.7)	
**Deaths, n (%)**	44 (20.6)	20 (12.8)	33 (43.4)	**<0.001**
**Time to death, median (IQR)**	32.0 (9.00-50.0)	28.0 (10.2-35.5)	7.00 (2.00-20.0)	**0.014**
**CD4 (count), median (IQR)**	68.0 (24.2-122)	66.5 (33.5-147)	17.5 (6.00-44.0)	**<0.001**
**Hb (g/dL), median (IQR)**	8.30 (7.03-10.5)	9.15 (7.88-10.4)	7.60 (6.20-9.80)	**<0.001**
**IL-1β**	1.7 (1.61 - 1.78)	1.9 (1.84 – 2.00)	1.94 (1.83 - 2.09)	**<0.001**
**IL-1RA**	2.17 (1.91 - 2.52)	2.23 (2.04 - 2.58)	3.15 (2.86 - 3.45)	**<0.001**
**IL-2**	1.74 (1.68 - 1.79)	1.91 (1.86 – 2.00)	1.83 (1.75 - 1.9)	**<0.001**
**IL-4**	1.55 (1.43 - 1.64)	1.83 (1.76 - 1.91)	1.63 (1.54 - 1.69)	**<0.001**
**IL-5**	1.36 (1.25 - 1.46)	1.67 (1.57 - 1.76)	1.34 (1.23 - 1.44)	**<0.001**

**IL-6**	2.28 (1.98 - 2.51)	2.33 (2.12 - 2.5)	2.82 (2.65 - 3.15)	**<0.001**
**IL-7**	1.46 (1.38 - 1.51)	1.66 (1.59 - 1.74)	1.46 (1.39 - 1.56)	**<0.001**
**IL-8**	1.96 (1.83 - 2.15)	2.08 (1.97 - 2.21)	2.58 (2.44 - 2.99)	**<0.001**
**IL-9**	2.08 (2 - 2.17)	2.28 (2.19 - 2.37)	2.36 (2.25 - 2.48)	**<0.001**
**IL-10**	1.74 (1.68 - 1.81)	1.94 (1.88 - 2.02)	1.88 (1.8 - 2.01)	**<0.001**
**IL-12p70**	1.62 (1.55 - 1.71)	1.91 (1.81 - 2.02)	1.65 (1.59 - 1.75)	**<0.001**
**IL-13**	1.48 (1.34 - 1.63)	1.72 (1.61 - 1.91)	1.43 (1.34 - 1.53)	**<0.001**
**IL-15**	1.88 (1.8 - 1.93)	2.02 (1.98 - 2.11)	2.06 (1.95 - 2.16)	**<0.001**
**IL-17**	1.69 (1.59 - 1.78)	2.00 (1.91 - 2.08)	1.77 (1.7 - 1.88)	**<0.001**
**Eotaxin**	1.72 (1.63 - 1.8)	1.91 (1.83 - 2.03)	1.88 (1.76 - 2.02)	**<0.001**
**FGF**	1.65 (1.57 - 1.71)	1.87 (1.79 - 1.94)	1.68 (1.61 - 1.73)	**<0.001**
**GCSF**	1.72 (1.63 - 1.81)	1.90 (1.82 - 1.99)	2.08 (1.91 - 2.4)	**<0.001**
**GMCSF**	1.87 (1.77 - 1.96)	2.02 (1.95 - 2.12)	1.97 (1.88 - 2.06)	**<0.001**
**IFN-γ**	1.59 (1.49 - 1.68)	1.88 (1.8 - 1.95)	1.76 (1.64 - 1.9)	**<0.001**
**CXCL10**	3.77 (3.51 - 3.99)	3.84 (3.54 - 4.06)	4.23 (4.12 - 4.35)	**<0.001**
**CCL4**	1.89 (1.8 - 1.99)	2.03 (1.94 - 2.15)	2.3 (2.14 - 2.62)	**<0.001**
**CCL2**	1.85 (1.71 - 2.08)	2.03 (1.91 - 2.21)	2.38 (2.13 - 2.6)	**<0.001**
**PDGF-bb**	1.97 (1.8 - 2.31)	2.65 (2.43 - 2.86)	1.91 (1.77 - 2.13)	**<0.001**
**CCL3**	2.77 (2.59 - 2.95)	2.81 (2.61 - 3.02)	3.4 (3.11 - 3.54)	**<0.001**
**CCL5**	4.15 (4.03 - 4.2)	4.2 (4.17 - 4.23)	4.01 (3.73 - 4.14)	**<0.001**
**TNF**	1.54 (1.47 - 1.61)	1.75 (1.69 - 1.84)	1.64 (1.57 - 1.72)	**<0.001**
**VEGF**	1.92 (1.8 - 2.02)	2.2 (2.09 - 2.34)	2.04 (1.91 - 2.18)	**<0.001**

**TGF-β1**	1.21 (1.07 - 1.46)	1.74 (1.53 - 1.9)	1.19 (1.13 - 1.4)	**<0.001**

Table note:

Bold font indicates statistical significance. Mild anemia was defined as Hb value >10 g/dL and <13 g/dL for men; and >10 and <12 g/dL for women, whereas moderate anemia was defined as Hb>8 g/dL and <=10 g/dL for both sexes. Severe anemia was defined as Hb<8g/dL for both sexes. Data are shown as median and interquartile range (IQR). The Kruskall-Wallis test was used to assess the statistical differences between all groups. The Cochran–Armitage test for trend was used to assess for the presence of an association between the measurements and the severity of anemia. Abbreviations: IQR: Interquartile range.
